# Developmental Changes in Hippocampal CA1 Single Neuron Firing and Theta Activity during Associative Learning

**DOI:** 10.1371/journal.pone.0164781

**Published:** 2016-10-20

**Authors:** Jangjin Kim, Mary E. Goldsberry, Thomas C. Harmon, John H. Freeman

**Affiliations:** Department of Psychological and Brain Sciences, University of Iowa, Iowa City, IA 52242, United States of America; University of Wisconsin-Milwaukee, UNITED STATES

## Abstract

Hippocampal development is thought to play a crucial role in the emergence of many forms of learning and memory, but ontogenetic changes in hippocampal activity during learning have not been examined thoroughly. We examined the ontogeny of hippocampal function by recording theta and single neuron activity from the dorsal hippocampal CA1 area while rat pups were trained in associative learning. Three different age groups [postnatal days (P)17-19, P21-23, and P24-26] were trained over six sessions using a tone conditioned stimulus (CS) and a periorbital stimulation unconditioned stimulus (US). Learning increased as a function of age, with the P21-23 and P24-26 groups learning faster than the P17-19 group. Age- and learning-related changes in both theta and single neuron activity were observed. CA1 pyramidal cells in the older age groups showed greater task-related activity than the P17-19 group during CS-US paired sessions. The proportion of trials with a significant theta (4–10 Hz) power change, the theta/delta ratio, and theta peak frequency also increased in an age-dependent manner. Finally, spike/theta phase-locking during the CS showed an age-related increase. The findings indicate substantial developmental changes in dorsal hippocampal function that may play a role in the ontogeny of learning and memory.

## Introduction

Hippocampal maturation has been proposed to be an important factor in the ontogeny of learning and memory [1]. Most of the evidence for the hippocampal maturation hypothesis comes from behavioral and lesion studies showing developmental changes in acquisition and retention of hippocampus-dependent tasks [2,3]. Rodent studies have found that spatial delayed alternation and hidden platform localization in the water maze become robust around the third postnatal week [4–6]. Context conditioning and the context pre-exposure facilitation effect also develop around the third postnatal week in rats [2,7–10]. The ontogenetic emergence of these tasks and others suggest that the efficacy of hippocampal contributions to learning increases between postnatal day (P) 17 and 24 in rats. The strikingly consistent developmental time course of hippocampus-dependent learning suggests that there are developmental changes in hippocampal function during learning within the first several postnatal weeks in rodents. A pair of studies examined the development of hippocampal place cells during exploration of an apparatus, but there were no learning contingencies in these studies [11,12]. It is therefore critical to examine developmental changes in hippocampal function during learning to elucidate the mechanisms underlying the ontogeny of learning and memory.

The current study examined the development of hippocampal physiological properties in rat pups during an associative learning task, eyeblink conditioning. Eyeblink conditioning involves paired presentations of a conditional stimulus (CS), such as a tone, and an unconditional stimulus (US), such as periorbital stimulation, resulting in the development of an eyelid closure conditional response (CR). This associative learning task depends on the cerebellum and interconnected brainstem nuclei [13,14]. Septohippocampal theta modulates the rate of acquisition in adult animals, possibly through attentional mechanisms [15–18]. Pre-training levels of theta activity accurately predict the rate and magnitude of delay eyeblink conditioning in adult rabbits [15,17,19]. Moreover, when adult rabbits are trained with theta-contingent trial presentations, they learn faster than yoked controls [17]. Hippocampal theta becomes synchronized with cerebellar theta during eyeblink conditioning and this synchronization may facilitate cerebellar plasticity and thereby facilitate learning [20,21]. The modulatory role of the hippocampus in eyeblink conditioning is also evident in dorsal hippocampal neuronal activity, which is influenced by pacemaker neuron populations of the medial septum [22]. A number of studies have found correlations between hippocampal spike activity and eyeblink conditioning [23–27]. Learning-related changes in dorsal hippocampal CA1 activity occur very early in learning. An increase in firing is seen during the US period that is associative and precedes the emergence of the CR across training trials. As training progresses, the CR is acquired and CA1 activity develops during the CS period. The firing rate of CA1 neurons (single and multi-unit clusters) correlates with the amplitude and time course of the CR and precedes the CR by approximately 40 ms [24,25]. The rapidly developing learning-related activity in CA1 neurons suggests a role for hippocampal neurons in determining the significance of external stimuli and how well they are encoded [28–30]. The medial septum receives direct inhibitory projections from the CA1 region of the hippocampus [31] and it has been suggested that this feedback from CA1 neurons to the medial septum may serve as a form of self-regulation for the hippocampus as the US becomes more precisely predicted by the CS [29,30].

Eyeblink conditioning becomes progressively stronger between P17 and P24 in rats [32,33], and therefore develops during the period in which spatial memory and context conditioning develop. A previous study found that lesions of the medial septum impair acquisition of eyeblink conditioning in rat pups trained on P21-23 or P24-26, but not in pups trained on P17-19, suggesting that septohippocampal modulation of eyeblink conditioning emerges ontogenetically between P19 and P21 [34]. However, another study suggested that hippocampal function in associative learning might continue to develop past P21 [35]. The current study further examined the development of hippocampal modulation of eyeblink conditioning by examining theta and single-neuron activity in the dorsal hippocampal CA1 region while rat pups were trained in eyeblink conditioning on P17-19, P21-23, and P24-26. Training began with an unpaired session (UP) that allowed us to investigate possible developmental changes in hippocampal theta and neuronal responsiveness to the CS and US before associative training. Following unpaired training, pups received 5 sessions of CS-US paired training, which in older pups, results in high levels of learning. The development of the CR was analyzed and compared to theta (4–10 Hz) activity, CA1 pyramidal neuron activity, and interactions between the two measures.

## Materials and Methods

### Subjects

The subjects were 21 male and female Long-Evans rat pups, counterbalanced for sex and condition, and trained on P17-19 (n = 7), P21-23 (n = 8), or P24-26 (n = 6). All rats were given *ad libitum* access to water and food and maintained on a 12 h day/night cycle. Pups trained on P21-23 or P24-26 were weaned on P19 and housed with littermates. Pups in the P17-19 group were returned to the dam and littermates following surgery and between training sessions. Experimental groups included no more than two pups from the same litter (one male and one female). All training sessions were conducted during the light cycle. All training and surgical procedures were approved by the Institutional Animal Care and Use Committee at the University of Iowa.

### Surgery

Two days before training, subjects were anesthetized with isoflurane (1–4%) for surgery. A recording drive with five tetrodes (one reference and four recording) was implanted above the right dorsal hippocampus (AP: -3.7 ~ -4.0 mm; ML: -2.3 ~ 2.5 mm depending on age group) for the electrophysiological recording of neuronal signals. Any space between the drive and skull was sealed with silicon (Kwik-Sil; World Precision Instruments). In addition, stainless-steel electromyography (EMG) electrodes for monitoring eyelid movement were implanted into the upper left orbicularis oculi muscle. The EMG ground was secured to the skull slightly anterior to the bregma. The EMG electrodes and ground wire terminated in gold pins inserted into a plastic connector strip. A bipolar stimulating electrode for delivering the US was implanted subdermally, caudal to the left eye. Bone cement (Zimmer) was used to secure the EMG and bipolar electrodes as well as recording drive to the skull. Immediately following surgery, all of the tetrodes were lowered approximately 1000 μm into the brain and pups were placed on a heating pad until fully recovered.

### Conditioning Apparatus and Procedures

**Fig 1:**
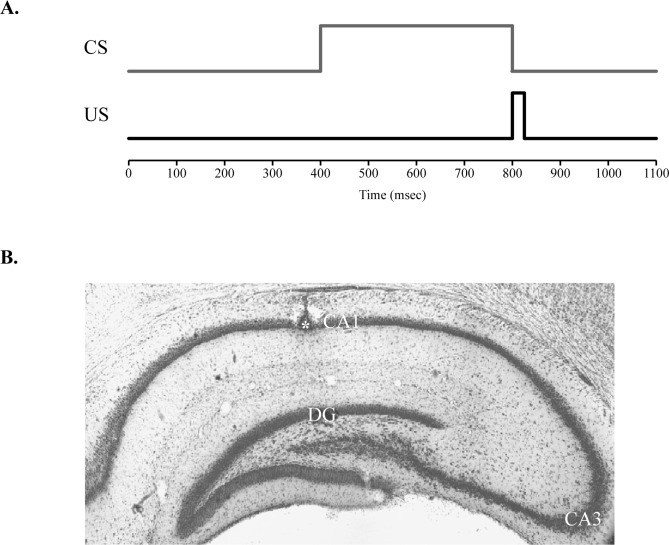
Delay eyeblink conditioning procedure and tetrode placement. **A.** Diagram of the delay conditioning procedure used in the current study. The time course of the conditioned stimulus (CS; tone) and the unconditioned stimulus (US; shock) during paired sessions are indicated by gray and black lines, respectively. Both the baseline period and CS period are 400 ms. US and post-US periods are 25 and 275 ms, respectively. Each paired session consisted of 100 trials equally divided into 10 blocks. **B.** Representative tetrode placement in the CA1 cell field of the dorsal hippocampus. Asterisk indicates tetrode tip position.

Detailed information regarding the conditioning apparatus can be found in previous publications [32,35,36]. Briefly, the conditioning chamber (BRS/LVE) was contained within a sound attenuation chamber. The CS tone (2.0 kHz, 85 dB, 400 ms) was delivered through a speaker on one side of the conditioning chamber. The US periorbital shock (2–3 mA, 25 ms duration) was delivered by a stimulus isolator (model number 365A; World Precision Instruments). Unpaired training consisted of 100 CS-alone and 100 US-alone trials separated by a 15 s intertrial interval. The stimuli were presented in a pseudorandomly shuffled order. During paired training, the US immediately followed CS offset (Fig 1A). Paired sessions were composed of 90 paired trials and 10 CS-alone probe trials (total 100 trials). The CS-alone trials were presented in every 10^th^ trial. The intertrial interval for paired sessions averaged 30 s (20–40 s range). Both unpaired and paired training sessions lasted approximately 1 hour. Presentation of stimuli and recording of eyelid EMG activity was controlled by computer software (JSA Designs). Differential EMG activity was filtered (500–5000 Hz), amplified (x 2000), and integrated for later analyses. Beginning two days after surgery, the pups received two training sessions per day for three continuous days, for a total of six sessions. Sessions that occurred on the same day were separated by 4 hours. The first session was unpaired (UP). The following 5 sessions were paired (S1-S5).

EMG signals that were 0.4 units above baseline activity during the CS, but not within the 80 ms startle period, were regarded as a CR. Trials with EMG activity that crossed the threshold prior to CS onset were omitted from analyses. The amplitude, onset latency, and peak latency of the CR were measured from CRs that occurred on CS-alone trials to avoid UR contamination.

### Recording Setup

Nichrome wires (12 μm in diameter; Kanthal) were twisted and heat-fused to form a tetrode. One end of the wires fed into an electronic interface board while the other end was implanted into the brain. Final impedance of each wire was adjusted to 200–300 kΩ (impedance tester IMP-1; Bak Electronics) by gold plating the tips (SIFCO Process) before implantation. The electronic interface board was connected and the output signals were amplified by 10,000–35,000 times and bandpass-filtered (0.1–6.0 KHz). Neural signals that passed an amplitude threshold were digitized and stored at 32 kHz (Cheetah System; Neuralynx).

On the day after surgery, recording tetrodes were further adjusted while electrophysiological properties of spikes were monitored as they approached the hippocampal CA1 area. Each adjustment was not greater than 40 μm and a 15 min rest interval was given after each adjustment. Before the experimental recording, a 30 min rest interval was given to obtain stabilized signals.

### Spike Isolation and Criteria

Spikes were isolated offline using MClust (MClust 4.1; A. D. Redish, University of Minnesota, Minneapolis, MN). Peak, valley, energy, and principal components of spike waveforms were used to separate the activity of individual neurons. Each neuron was further assessed with parameters such as waveform, interspike interval, autocorrelation, firing rate, number of spikes, stability, l-ratio, and isolation distance. Spike activity was categorized as pyramidal neuron activity by assessing histology and physiological parameters. Only neurons recorded from the CA1 layer of the hippocampus were used (Fig 1B). The neurons were further filtered to meet criteria of (1) a bimodally distributed interspike interval histogram, (2) more than 100 spikes in session, (3) greater than 0.5 Hz firing rate, (4) more than 250 μs spike width, (5) smaller than 0.5 l-ratio, and (6) greater than 8.0 isolation distance were used to have complex spike neurons only for the analyses.

### Neuronal Data Analysis

All neuronal data analyses were done with customized Matlab code. Spectral analyses of local field potentials (LFPs) were done with the Chronux Toolbox (http://www.chronux.org). The Circular statistics toolbox for Matlab was used for spike/phase locking analyses and statistical tests [37].

### Population PETH Map

To visually assess neuronal firing patterns during training, population perievent time histogram (PETH) maps were constructed for each session and age group [38]. Spike trains of neuronal populations were sorted by the maximal peak position within trials. The firing rate of each neuron was normalized to range between 0 and 1. If neuronal firing is not specifically bound to a certain event, the population PETH map would show diagonally aligned maximal firing peaks in maps. However, if neurons show event-related firing, the arrangement of the peaks would be curved either downwardly or upwardly in maps.

### Perievent Time Histograms

Changes in hippocampal CA1 neuronal firing were examined in PETHs. Spikes from the pre-CS baseline period through the post-US period were sorted in trial-by-trial format using a 100 ms bin resolution. Both CS and US onsets were trial events of interest. The number of spikes in each PETH was z-transformed to determine single unit trial event-responsiveness as well as for the population analysis. The z-score was calculated by dividing the difference between number of spikes in each bin and the confidence mean by the square-root of the confidence mean. The confidence mean was the expected mean bin value with the alpha level of 0.05. Either a Poisson distribution or a Gaussian distribution was assumed in calculating the confidence mean, depending on how many spikes existed in each bin (i.e., Gaussian distribution for more than 30 Hz and Poisson distribution for less or equal to 30 Hz, respectively). If the z-scores of the bins exceeded the confidence limits (Bonferroni corrected alpha = 0.00625), the neuron was regarded as event-responsive. Depending on which bins were above threshold, neuron was regarded as event-responsive. Also, the neurons were further sub-typed as either CS-responsive or post-US responsive or both-responsive [32,35,36]. Only the task-responsive neurons were used in the following spike analyses.

### Spectral Analysis of LFPs

The LFP analysis was done on a single tetrode (of four available) that had the least adjustment before the session as well as the largest number of neurons [39,40]. Raw LFPs were downsampled from 32 kHz to 1894 Hz for easier data handling. Then, LFPs were sorted into a trial-by-trial format using the timestamp information corresponding to trial events. Theta LFPs (4–10 Hz) were obtained by bandpass-filtering raw LFPs. For each trial, a spectral analysis was done to determine significant theta power changes that occurred during the CS. The theta power values between the pre-CS and CS periods were statistically compared using a nonparametric Wilcoxon signed-rank test (alpha = 0.01). In every trial, the theta power value during each event was measured by calculating the spectral power ratio between theta and theta + delta (1–4 Hz); power values within the theta frequency range were summed and then divided by the summed power values from the broader band (1–10 Hz). The number of trials with significant theta power change, theta ratio, and the theta peak frequency were used as dependent measures [21].

### Phase Synchrony between Spike Trains and Theta LFPs

The theta phase information during trials was obtained by applying a Hilbert transformation to the bandpass-filtered LFPs [41]. Spike phase histograms (20° bin size) were generated for each session. Rayleigh’s circular statistical test (alpha = 0.05) was done to assess whether neuronal activity was significantly locked to certain phase of theta. The proportion of statistically significant neurons in each session, as well as in each age, was used as the dependent measure.

### Histology

Electrolytic lesions were made after the completion of the experiment to verify tetrode tip positions in the brain (10 μA, 8 s). The following day, rat pups were anesthetized with sodium pentobarbital then transcardially perfused with saline and 10% buffered formalin. Brains were placed in 30% sucrose-formalin for cryoprotection. Brains were then frozen, sectioned at 50 μm, mounted on glass slides, and stained with thionin for recording site verification under a light microscope.

## Results

### Histology and Neuronal Classification

After the completion of training, recording sites were histologically verified (Fig 1B). Only neurons recorded from the CA1 layer of the dorsal hippocampus were used. Recordings from the dentate gyrus or other areas were omitted from the initial filtering. As noted in the methods, other physiological properties were examined for further filtering to prevent the inclusion of theta cells or interneurons. Overall, 1401 neurons were initially obtained from 20 rat pups. After filtering with physiological parameters, 843 pyramidal neurons remained (Table 1). Descriptive statistics of included pyramidal neurons are as follows: Median number of spikes = 6067, mean firing rate = 3.18 Hz (range: 0.50 ~ 31.23 Hz), mean spike width = 364.37 μs, median of l-ratio = 0.27, and median isolation distance = 14.17. These values were similar between unpaired and paired sessions.

**Table 1:** Count, the median number of spikes, and the mean firing rate in recording session of pyramidal neurons used in analyses.

	UP	S1	S2-3	S4-5
**P17-19**	67 (5200; 2.23 Hz)	22 (3759; 2.21 Hz)	40 (3309; 1.37 Hz)	47 (2254; 1.17 Hz)
**P21-23**	93 (7460; 3.34 Hz)	105 (5754; 2.72 Hz)	104 (5599; 2.98 Hz)	81 (4234; 2.29 Hz)
**P24-26**	75 (6587; 3.31 Hz)	73 (5613; 3.65 Hz)	81 (4358; 2.73 Hz)	55 (6618; 2.73 Hz)

### Developmental Changes in Eyeblink Conditioning

**Fig 2:**
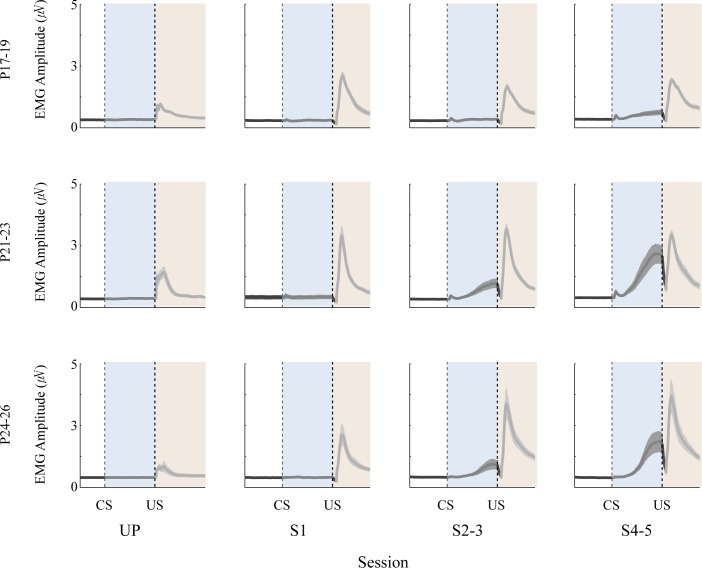
Population mean (+/- SE) eyelid EMG traces for rat pups given delay eyeblink conditioning. The vertical dotted lines indicate CS and US onsets. Note that responses during the CS and US in the unpaired (UP) session were plotted together for visualization. EMG amplitude during the CS period increased across paired sessions (S1, S2-3, and S4-5) in all age groups. However, these increases were more evident in the older two age groups. The drop in EMG activity between the CS period and the post-US period is caused by gating the amplifier during the US to prevent recording a stimulation artifact.

**Fig 3:**
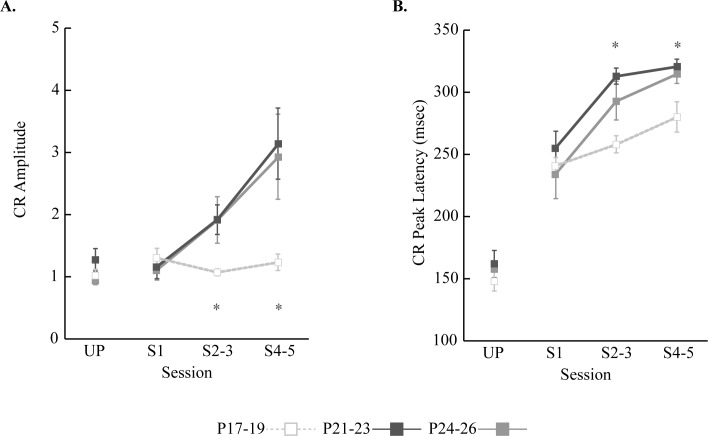
Mean (+/- SE) eyelid conditioned response (CR) amplitude and CR peak latency across the unpaired (UP) and paired sessions (S1-S5) for rat pups trained on postnatal days (P) 17–19, P21-23, or P24-26. **A.** Mean CR amplitude. The two oldest age groups showed greater increase in CR amplitude across paired sessions than the youngest age group. **B.** Mean CR peak latency. During UP, eyelid responses tended to occur 150 ms after the CR onset. However, as paired training progressed, CR peak latency was more aligned toward the US onset in all age groups. The changes were more evident in the two older age groups than P17-19.

Eyelid EMG activity was measured and plotted across training sessions for each age group (Fig 2) and statistically tested with the CR amplitude, CR percentage, and CR peak latency variables (Fig 3). CR amplitudes were comparable during both UP and S1 in all age groups. As paired training continued, the two older age groups showed linear CR amplitude increases while the P17-19 group remained unchanged. A repeated-measures ANOVA with session and age group as factors confirmed these observations. There were an interaction of age group and session (*F*_(6, 54)_ = 3.73, *p* < 0.01). *Post-hoc* pairwise comparisons were conducted using a modified Benjamini-Hochberg false discovery rate procedure [42,43]. This method was used for all *post-hoc* pairwise comparisons in repeated-measures ANOVA. The tests showed that the CR amplitudes in the P17-19 group did not differ across sessions (*p*s > 0.09), whereas the two older age groups showed significant increases across training sessions (*ps* < 0.05). Across age groups, CR amplitude was similar during UP and S1 (*p*s > 0.09), but diverged during S2-3 and S4-5 (*p*s < 0.05). The two older age groups showed significantly higher CR amplitudes than the P17-19 group. Comparisons between the two older age groups were not significant (*p*s > 0.09) (Fig 3A).

As observed in the CR amplitude data, there was an age-related increase in CR percentage. A repeated-measures ANOVA on the CR percentage data demonstrated an interaction of age group and session (*F*_(6, 54)_ = 5.16, *p <* 0.001). *Post-hoc* comparisons indicated that CR percentage in all age groups increased significantly during S2-3 compared to UP (*p*s < 0.05). The comparisons across the age groups showed that CR percentage in the P17-19 group was higher than the two older age groups during both UP and S1 (*p*s < 0.05). However, from S2-5 the CR percentage in the older age groups was significantly higher than the P17-19 group (*p*s < 0.05). CR percentage was similar between the two older age groups throughout training (*p*s > 0.07).

CR peak latency also showed developmental and learning-related changes across training. CR peak latency was around 160 ms during UP training and then increased across sessions in all the age groups, thus suggesting improved timing of the CR. Importantly, CR peak latency increased more in the two older age groups than in the P17-19 group during training sessions S2-3 and S4-5. A repeated-measures of ANOVA showed significant main effects of age group (*F*_(2, 18)_ = 7.13, *p* < 0.01) and session (*F*_(3, 54)_ = 141.27, *p* < 0.001). However, the interaction between the two factors was not statistically significant (*F*_(6, 54)_ = 1.62, *p* = 0.16). *Post-hoc* pairwise comparisons showed that CR peak latency in the two older age groups significantly differed from the P17-19 group during S2-3 as well as S4-5 (*p*s < 0.05) (Fig 3B). Overall, the observed changes in eyelid EMG activity were similar to those seen in previous studies [32,34].

### Baseline CA1 Spike Activity

The overall number of spikes and mean firing rates in each recording session were statistically tested in a repeated-measures ANOVA using age group and session as factors. There were no significant effects related to age group, session, or an interaction (*F*s < 0.61, *p*s > 0.69). These results indicate that there were no differences in baseline activity between age groups or across sessions.

### Developmental Changes in the Distribution of CA1 Neurons Responsive to Trial Events

**Fig 4:**
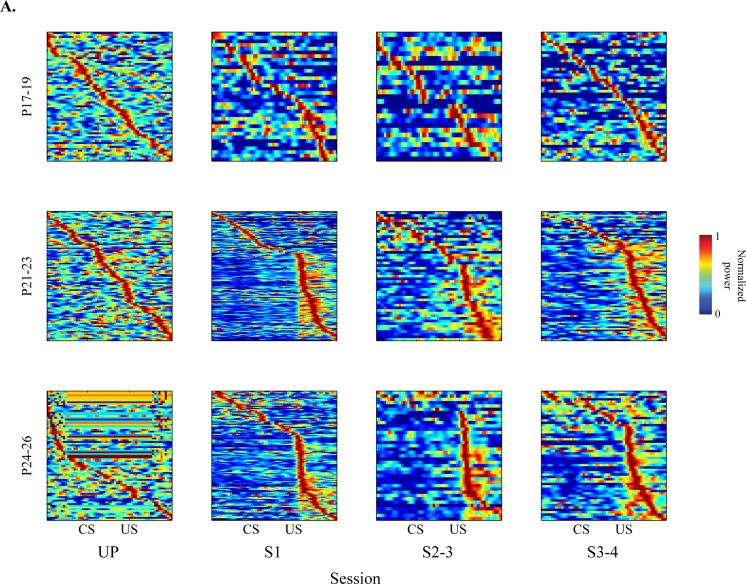
Population maps of CA1 firing. **Population PETH maps across the sessions for rat pups given eyeblink conditioning on postnatal days (P) 17–19, P21-23 or P24-26.** Population PETH maps were constructed by sorting the neurons by peak firing time positions to show the distribution of the maximal neuronal firing in each session and age group. For visualization, the firing rate for each neuron was individually normalized and scaled from 0 to 1. Neurons recorded from the P17-19 group showed linearly aligned patterns of peak firing across the sessions indicating that the neurons did not show specific event-related firing. However, both P21-23 and P24-26 groups showed upwardly curved patterns of peak neuronal firing during paired sessions indicating that maximal firing rates were highly concentrated in the post-US period.

Population PETH maps (10 ms/bin) were generated to visually assess the distribution of pyramidal cell categories in each age group across training sessions [38]. Neurons in each session were sorted along the peak firing position in time (Fig 4). If neuronal activity was not preferentially responsive to trial events, the sorted maximal firing peaks would appear as a straight diagonal line across the map. If, on the other hand, neurons were preferentially responsive to trial events, peak activity would be distributed along a non-linear curve. During the unpaired session, population PETH maps in both the P17-19 and P21-23 groups showed a linear pattern of maximal firing peaks, indicating that the activity was similarly distributed during trials. However, in the P24-26 group, the peaks were distributed in a downward curved pattern, suggesting that neurons showed more activity during the pre-CS baseline period. During paired training, population PETH maps of peak activity in the P17-19 group remained linear. In both the P21-23 and P24-26 groups, however, maximal activity was concentrated in the post-US period (Fig 4). These results indicate that many of the CA1 neurons become post-US responsive with CS-US training in the older groups, but not in the P17-19 group.

**Fig 5:**
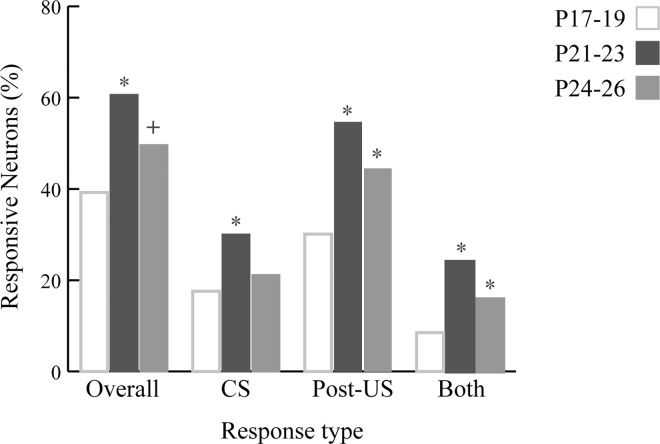
The distribution of response types of CA1 pyramidal cells in rat pups. The response types were categorized as Overall, CS, Post-US, and Both responsive. The P21-23 group showed the highest proportion of responsive neurons in all conditions. The oldest age group had more responsive neurons than the youngest in the Overall, Post-US, and Both conditions. The neurons in the youngest group were less responsive to the post-US period, compared to the older age groups. Asterisks represent the statistical significance of alpha < 0.05. Green cross indicates alpha = 0.05.

In order to determine whether individual neurons showed firing rate changes in response to specific trial events, individual hippocampal CA1 pyramidal cell activity was assessed in PETHs (100 ms/bin; Fig 5). Spike activity was plotted and compared to baseline across two different 400 ms periods: the CS- and post-US periods (Bonferroni corrected alpha = 0.00625)[35,36]. Neurons were categorized according to the trial event to which they were responsive (either CS- or post-US responsive). Thus, a neuron could be categorized as Overall-responsive (showing responsiveness to any event or combination of events), CS-responsive, post-US responsive, or Both (CS and US)-responsive. In the categories of Overall-responsive, Post-US responsive, and Both-responsive, the two older age groups showed higher proportions of responsive neurons than the P17-19 group. When examining CS-responsive neurons, the P21-23 group showed a higher proportion than the other two age groups; the P17-19 and P24-26 age groups were comparable to each other. Chi-square tests confirmed these observations (all *ps* < 0.05). These results suggest that hippocampal CA1 neurons in the two older age groups were more responsive to trial events than the P17-19 age group (Fig 5).

### Developmental Changes in the CA1 Firing Rate

**Fig 6:**
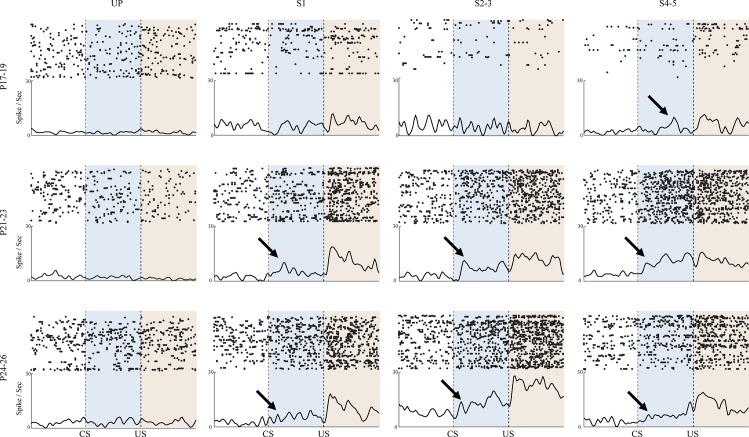
Representative examples of CA1 pyramidal cell firing. Examples of CA1 single pyramidal cell firing during eyeblink conditioning in rat pups given eyeblink conditioning on postnatal days (P) 17–19, P21-23, or P24-26. Rastergrams and perievent time histograms (PETH; bin size = 10 ms) of single neurons recorded during the unpaired (UP) session and following paired sessions (S1, S2-3, and S4-5). The neurons in the P17-19 group tended to fire randomly across the CS and US presentations until S2-3. In contrast, neurons in the older age groups showed increased firing rates during the post-US period as early as S1 and showed increased firing rates in the CS and post-US period as early as S2-3. Arrows note the start of significantly increased activity during the CS.

During training the neurons in the P17-19 group did not show much specific event-related firing modulation (Fig 6, top row). However, the neurons in the older two age groups showed substantial event-related firing during the CS and post-US periods (Fig 6, middle and bottom rows). Specifically, during UP training neurons in all three age groups randomly fired across the trial events. This firing pattern was maintained in the P17-19 group. However, the firing rates were selectively increased during the CS and post-US periods in both the P21-23 and P24-26 age groups during CS-US paired training.

**Fig 7:**
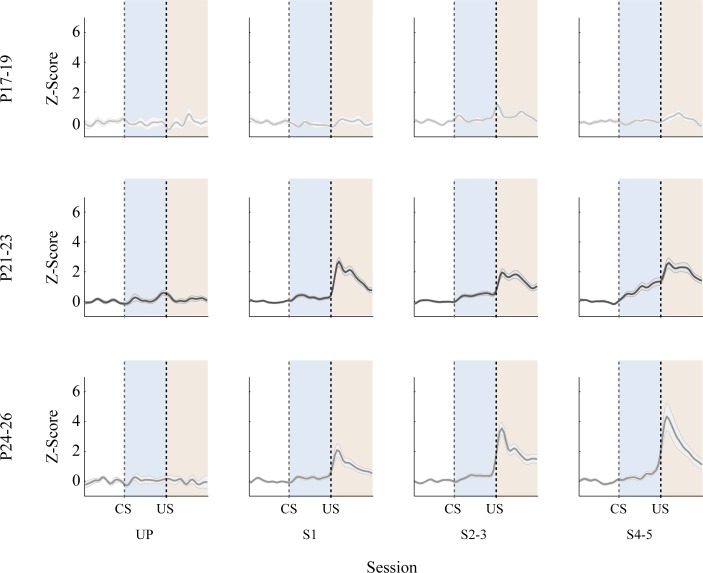
Mean (+/- SE) CA1 pyramidal cell firing rate changes in rat pups given delay eyeblink conditioning on postnatal days (P) 17–19, P21-23, or P24-26. During the unpaired (UP) session, firing rates were comparable during the stimuli across age groups. Once paired sessions (S1, S2-3, and S4-5) started, however, neurons in the older age groups showed greater activity during both the CS and post-US periods. The differences between the P17-19 group and the older groups became larger with the training, especially in the post-US period. Firing rates between the P21-23 and P24-26 groups were comparable.

Using task-responsive neurons only, population mean firing rates of hippocampal CA1 pyramidal neurons were assessed across sessions as well as age groups (Fig 7). As seen with single neurons, the firing rates in the P17-19 group did not change in relation to the CS or US. Conversely, the firing rates in both the P21-23 and P24-26 groups showed sharp increases during the CS and post-US periods with paired training. Z-scores in bins (20 ms/bin) from the three age groups were statistically tested using repeated-measures ANOVA, yielding a significant bin, age group, and session interaction (*F*_(354, 25901)_ = 1.99, p < 0.001). *Post-hoc* pairwise comparisons showed that there was greater trial event-related firing in the two older age groups than in the P17-19 group (*p*s < 0.001). The results were the same throughout the paired training sessions (*p*s < 0.001). Z-scores in UP training were not different across age groups (*p*s > 0.61).

### Developmental Changes and Learning Effects on Hippocampal Theta

**Fig 8:**
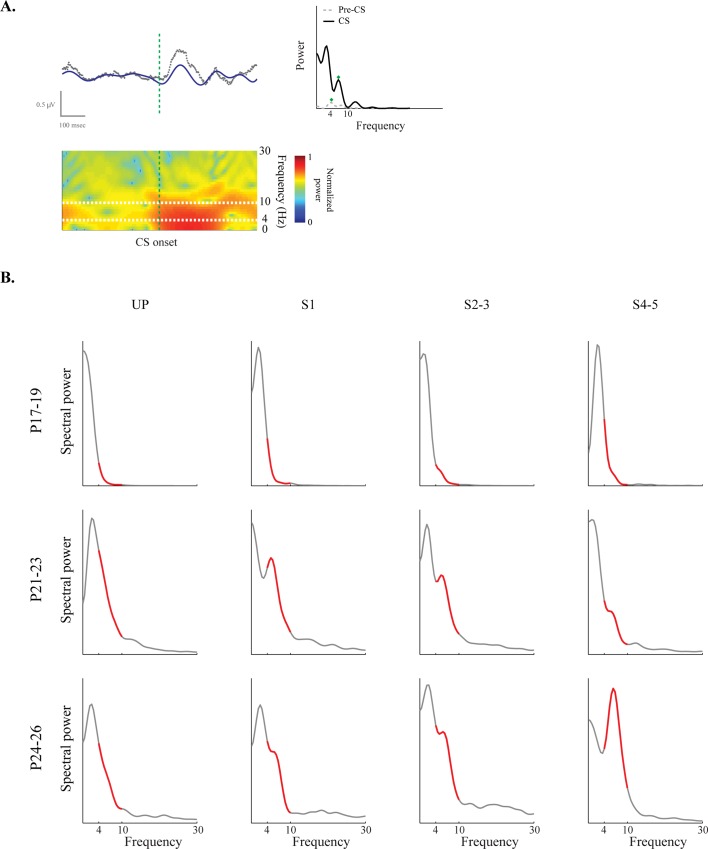
Power analyses of hippocampal theta in rat pups given delay eyeblink conditioning on postnatal days (P) 17–19, P21-23, or P24-26. **A.** Schematic illustration of the trial-by-trial analysis of theta rhythm. Gray and blue LFPs indicate the raw activity and theta (4–10 Hz) rhythm in a trial, respectively. Raw LFP in a given trial (gray) was bandpass filtered to obtain the theta LFP (blue). Both spectral periodogram (right) and spectrogram (bottom) were generated to compare theta power changes across the CS. Horizontal and vertical axes of the scale represent time and LFP amplitude, respectively. Vertical dotted lines indicate the CS onset. Green diamonds on the periodograms indicate the theta peaks in the event periods. White dotted lines on the spectrograms denote the theta range. **B.** Representative examples of spectral power density functions from a single rat pup in each age group. Spectral power was observed and drawn across each training session for all age groups. Gray and red lines indicate the spectral power in 1–30 frequency range and theta frequency range (4–10 Hz), respectively. It is noteworthy that theta ratio values (theta / (theta + delta (1–4 Hz)) were used for the population analyses, not the raw theta values.

Previous studies found relationships between theta power and associative learning in adult animals (Berry & Thompson, 1978; Hoffman & Berry, 2009; Nokia et al., 2008; 2012). It was therefore critical to examine developmental changes in pre-CS and CS-related theta in the current study. Theta power modulation during the CS was assessed across sessions as well as age groups to examine developmental changes in the relationship between theta power and learning (Fig 8). Raw LFPs in each session were bandpass-filtered into theta LFPs (4–10 Hz). Theta LFPs were parsed into a trial-by-trial format and power spectral analyses were conducted for each trial. Theta power during the pre-CS baseline period was statistically compared to the CS period (Fig 8A). In addition, theta power was calculated by determining the ratio between theta and a broader frequency range (theta + delta; 1–10 Hz) during trials (Fig 8B)[21].

**Fig 9:**
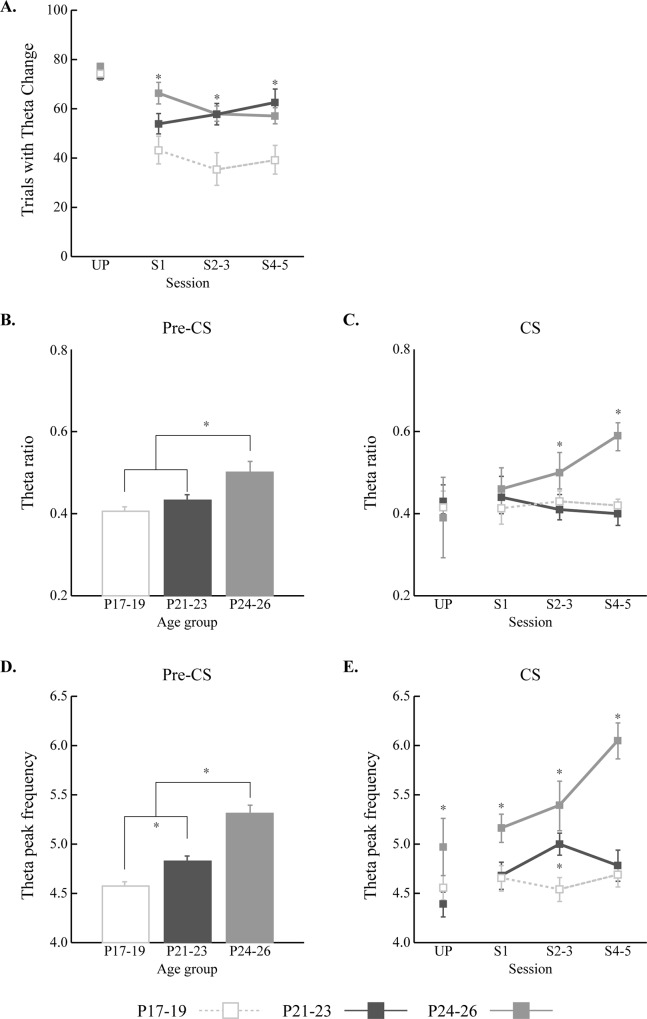
Hippocampal theta changes in rat pups given delay eyeblink conditioning on postnatal days (P) 17–19, P21-23, or P24-26. **A.** Mean (+/- SE) number of trials with a significant change in theta during the CS across sessions. The two older groups had more trials with significant changes in theta power than the P17-19 group in most paired sessions. **B and C.** Mean (+/- SE) theta ratio (theta / (theta + delta (1–4 Hz)) was calculated for both pre-CS and CS periods and compared across the training sessions and age groups. Theta ratio in the P24-26 group was higher than in the younger two age groups during the pre-CS and CS periods. **D and E.** Mean (+/- SE) theta peak frequency was assessed for both pre-CS and CS periods as well. In every spectral density function, the peak frequency value within the theta range was calculated to assess the theta shift across development and training. Theta peak frequency was distributed in age-dependent manner during the pre-CS.

The proportion of trials with significant theta power modulation during the CS was also calculated. During UP, the number of trials with significant theta power modulation was similar across age groups. Once paired training began, however, there were more trials showing significant theta power modulation in the older age groups (Fig 9A). A repeated-measures ANOVA with session and age group as factors showed an interaction of session and age group (*F*_(6, 51)_ = 2.64, *p* < 0.05). Among the age group comparisons, *post-hoc* tests showed that the P24-26 group had more trials with significant theta change across the CS than the P17-19 group (*p*s < 0.05). The results were similar when comparing the P21-23 and P17-19 groups (*p*s < 0.05), except during S1 (p = 0.11). The comparisons between the P24-26 and P21-23 groups were not significant across the sessions (*p*s > 0.08). None of the age groups showed significant changes across the paired sessions (*p*s > 0.11).

Changes in theta power were also examined across sessions and age groups (Fig 8B). To separate the developmental and learning effects on the theta power, analyses were done separately for the pre-CS and CS periods. Also, for population analyses, theta ratio within the theta + delta frequency range (1–10 Hz) was calculated and statistically compared. The theta ratio during the pre-CS period showed age-dependent differences, with the P24-26 group showing a higher theta ratio than the other two younger age groups (Fig 9B). A one-way ANOVA with age group as a factor confirmed this observation with a main effect of age group (*F*_(2, 123)_ = 6.40, *p* < 0.01). *Post-hoc* pairwise comparisons using Tukey HSD indicated that the main effect primarily came from the difference between the P24-26 group and the other two groups (*p*s < 0.05) rather than the differences between the P17-19 and P21-23 groups (*p* = 0.55) (Fig 9B). The theta ratio during the CS period was also examined (Fig 9C). Theta ratio values were higher in the P24-26 than the two younger groups (*F*_(2, 114)_ = 3.18, *p* < 0.05).

Theta peak frequency was examined across training sessions as well as between age groups. During the pre-CS period, theta peak frequency was distributed in an age-dependent manner (Fig 9D). A one-way ANOVA showed that there was a significant main effect of age group (*F*_(2, 123)_ = 29.50, *p* < 0.001). *Post-hoc* pairwise comparisons using Tukey HSD showed that theta peak frequency increased linearly across the age groups (*p*s < 0.05). (Fig 9D). Theta peak frequency in the CS period during UP was higher in the P24-26 group relative to the two younger groups and increased across paired training in this group (*F*_(6, 114)_ = 2.55, *p* < 0.05).

### Developmental Differences in Phase Synchrony between Theta LFPs and CA1 Spike Activity

**Fig 10:**
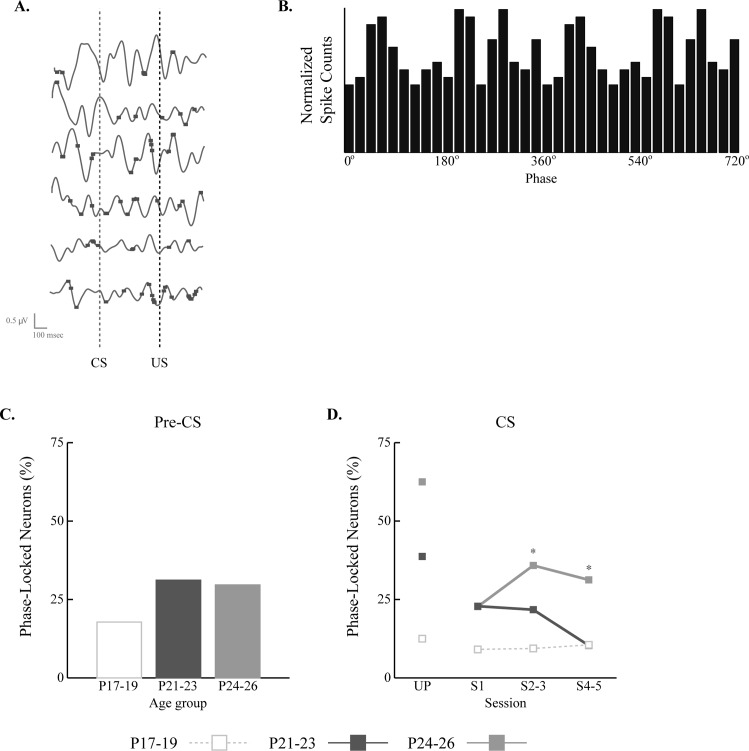
CA1 spike-theta phase relationships. **A.** Representative examples of theta and simultaneously recorded CA1 firing from a session. Horizontal and vertical axes of the scale represent time and the amplitude level of the EEG, respectively. **B.** The distribution of firing phases during the cycles of theta. The statistical significance of the distribution was tested with Rayleigh’s circular data test (alpha = 0.05). **C and D.** Percentage of neurons with significant phase-locking in rat pups given eyeblink conditioning on postnatal days (P) 17–19, P21-23 or P24-26 in the pre-CS and CS periods. The proportion of CA1 neurons that showed significant phase-locking with theta showed both developmental and learning effects.

Phase synchrony between CA1 spike activity and theta LFPs was calculated in the pre-CS and CS periods, separately (Fig 10). For each trial, phase information of the theta LFP was obtained by the Hilbert transformation. The distribution of CA1 spiking within theta phase was obtained for each cell and each distribution was statistically tested with Rayleigh’s circular statistical test. The percentage of neurons with significant phase locking was then calculated. In the pre-CS period, 26% of neurons showed significant phase-locking with ongoing theta rhythms. When broken down by age group, the two older age groups showed slightly higher levels (31% and 30% for the P21-23 and P24-26 groups, respectively) than the P17-19 group (18%), but the differences among the age groups were not statistically significant (*χ*^*2*^_(2)_ = 4.97, *p* = 0.08). During the CS period, neurons in the older two age groups showed more phase-locking to theta than the P17-19 group in most sessions and also they showed sharp decreases from UP to S1. However, while the oldest age group showed an increase in phase locking after S1, neurons in the P21-23 group showed a decrease. Chi-square tests showed that the differences among the age groups were not significant during UP (*χ*^*2*^_(2)_ = 4.24, *p* = 0.12) and S1 (*χ*^*2*^_(2)_ = 1.12, *p* = 0.57), but during S2-3 (*χ*^*2*^_(2)_ = 8.02, *p* < 0.05) and S4-5(*χ*^*2*^_(2)_ = 7.14, *p* < 0.05) the differences were significant. Subsequent comparisons of standard residuals indicated that the P24-26 group was significantly different from both the P17-19 and P21-23 groups during the later training sessions (*p*s < 0.05) (Fig 10). These results suggest that there is ongoing development in the rodent hippocampal neurons, which is related to phase-locked firing with the theta LFP.

## Discussion

The rate of associative learning increased as a function of postnatal age, as seen in previous studies [32,33]. Ontogenetic changes were evident in CA1 single neuron and theta activity. Greater task-related CA1 pyramidal cell activity was found in the older age groups (P21-23 and P24-26) relative to the youngest age group (P17-19). There were also developmental increases in the proportion of training trials with theta changes, the theta/delta ratio, theta peak frequency, and phase-locking between CA1 firing and theta oscillations (Table 2).

**Table 2:** Summary of behavior, CA1 firing, theta analyses and CA1-theta phase-locking results from three postnatal days (P) 17–19, P21-23, and P24-26 in delay eyeblink conditioning.

Measure		Age group comparisons
Behavior	CR amplitude (Figs 2 and 3A)	P17-19 < P21-23 ≒ P24-26
	CR percentage (Figs 2 and 3B)	
	CR percentage (Figs 2 and 3C)	
CA1 firing	PETH maps & distributions of the responsive cell types (Figs 4 and 5)	P17-19 < P24-26 ≤ P21-23
	CA1 cell firing rate (Figs 6 and 7)	P17-19 < P21-23 ≒ P24-26
Theta LFP	Significant theta power change (# trials) (Fig 9A)	P17-19 < P21-P23 ≒ P24-26
	Theta ratio (Fig 9B and 9C)	P17-19 ≒ P21-23 < P24-26
	Theta peak frequency (pre-CS) (Fig 9D)	P17-19 < P21-23 < P24-26
	Theta peak frequency (CS) (Fig 9E)	P17-19 ≒ P21-23 < P24-26
Phase-Locking of CA1 firing and theta LFP	Phase-lock (pre-CS) (Fig 10C)	P17-19 < P21-23 ≒ P24-26 (*p* = 0.08)
	Phase-lock (CS) (Fig 10D)	P17-19 ≒ P21-23 < P24-26

Hippocampal single neuron activity in the rat pups trained on P21-23 and P24-26 during eyeblink conditioning was very similar to hippocampal activity recorded from adult rabbits in previous studies [23–26]. In adult rabbits, CA1 neurons do not show increased activity during unpaired presentations of the CS and US, but show increases in firing to the US during the first paired session, even before the CR starts to emerge. As conditioning progresses, CA1 activity in adult rabbits starts to develop during the CS periods and models the amplitude and time course of the CR [23–25]. We found that CA1 neurons recorded on P21-23 and P24-26 did not respond to the stimuli strongly during the unpaired pre-training session but showed robust responses to the US during the first training session (Figs 4–7). The neuronal responses to the US were already robust before CRs increased above unpaired baseline levels (Figs 6 and 7). The youngest age group did not show much learning-related CA1 activity. These developmental changes in CA1 single neuron activity indicate that hippocampal coding of task-relevant events of conditioning continues to change ontogenetically as associative learning develops, becoming adult-like by P21-23.

An ontogenetic change in the proportion of trials with significant theta modulation during CS was found in the current study (Fig 9A). This finding is generally consistent with the findings from adult rabbits insofar as theta activity is associated with better acquisition [19]. However, we did not see a developmental change in the relationship between pre-CS theta power and the rate of eyeblink conditioning. We found that pups that acquired eyeblink conditioning rapidly had higher initial pre-CS theta power during the unpaired session than rat pups that acquired slowly (data now shown). Unlike adult animals [19], however, this difference was not maintained when paired training started. The absence of a developmental change in pre-CS theta facilitation suggests that although pre-CS theta facilitates delay eyeblink conditioning in adults, it does not influence the ontogeny of associative learning. It is possible that there are procedural differences that could account for this failure to detect age-related changes in the facilitation of eyeblink conditioning by pre-CS theta. For example, in the adult rabbit studies the animals were restrained, whereas the rat pups in the current study were unrestrained. The rats are typically immobile during paired training due to fear conditioning, but restraint may provide a very different context that increases attention and/or arousal, resulting in stronger theta oscillations in the pre-CS period. Species differences do not account for the absence of a relationship between pre-CS theta power and the rate of eyeblink conditioning because a recent study showed this effect in adult rats [44]. Another possibility is that previous studies in adult animals recorded LFPs in the hippocampal fissure, whereas our recordings were in the pyramidal layer (to optimize pyramidal neuron spike recordings). Thus, the current study may have had relatively weaker theta LFPs which partially obscured our ability to see effects of pre-CS theta levels on acquisition in rat pups.

Additional spectral analyses on the LFPs showed that the theta ratio was higher in the P24-26 group than in the younger two age groups during the pre-CS period (Fig 9B) and CS period (Fig 9C). Although the proportions of the trials with significant theta power changes were similar between the two older age groups (Fig 9A), theta ratio values (theta / (theta + delta)) were similar between the two younger age groups (Fig 9B and 9C). It is possible that the differences in the two measures are due to developmental differences in delta power [45,46]. To address this concern, we conducted an analysis of delta power differences across age groups and training sessions, and found that there was neither an age group nor a session effect in delta power (*F*s < 1, *n*.*s*.; data now shown). In addition, theta peak frequency measures showed similar results (Fig 9D and 9E). The spectral analyses on LFPs indicate that further developmental processes unfold between the two older age groups.

Substantial developmental changes were found in CA1 firing (Figs 4–7) and theta LFPs (Fig 9). These results are consistent with earlier findings with other paradigms [2,4–8,10–12], indicating that there is substantial hippocampal maturation around the third postnatal week. Once the animal passes this maturational period, associative learning and some physiological properties of the hippocampus become more adult-like. The two older age groups in the current study showed similar associative learning across paired sessions and their CA1 activity resembled that found in adult animals [23–26]. However, the current results also suggest that the hippocampus continues to mature after the third postnatal week in terms of orchestrating its signals with the theta rhythm. First, the P24-26 group had more theta phase-locked neurons compared to the P21-23 group (Fig 10). These phase synchrony differences indicate that hippocampal coding of the timing of the US relative to the CS is processed more precisely as rats mature. This hypothesis could be behaviorally tested by using a task in which precise temporal prediction of the US is more difficult. Overall, the results of phase synchrony and theta LFP indicate that although the P21-23 and P24-26 groups showed equivalent associative learning, there are developmental changes in septohippocampal mechanisms for processing task-related events.

The age range over which we found developmental changes in hippocampal physiological properties related to eyeblink conditioning corresponds well to the developmental time course of other associative and spatial memory tasks. Delayed alternation, water maze platform localization, contextual fear conditioning, and the context pre-exposure facilitation effect become robust after the third postnatal week in rats [2,5–7,9,10]. Earlier research from our laboratory also indicate that eyeblink conditioning continues to develop after the third postnatal week [32,35,47]. Overall, findings from spatial memory and fear conditioning studies, as well as the previous and the current eyeblink conditioning study, suggest that hippocampal learning mechanisms come online around the third postnatal week in rats. Therefore, the development of hippocampal firing, theta activity, and their orchestration may be a common mechanism underlying the ontogeny of multiple types of memory.

The current findings demonstrate that there are substantial developmental changes in hippocampal coding of associative learning. Developmental changes in CA1 spike activity, theta oscillations, and their interactions during eyeblink conditioning indicate the development of hippocampal function is playing a critical role in the ontogeny of learning and memory. Our analysis of developmental changes in hippocampal function during a relatively simple associative learning task provides a foundation for examining ontogenetic changes in hippocampal function during more complex episodic memory tasks.

## References

[1] Stanton ME, Ivkovich Claflin D, Herbert J. Ontogeny of Multiple Memory Systems: Eyeblink conditioining in Rodents and Humans Oxford Handbook of Developmental Behavioral Neuroscience. Oxford University Press; 2009 pp. 501–526.

[2] Rudy JW, Morledge P. Ontogeny of contextual fear conditioning in rats: Implications for consolidation, infantile amnesia, and hippocampal system function. Behavioral neuroscience. 1994;108: 227–34. 10.1037//0735-7044.108.2.227 8037868

[3] Stanton ME. Multiple memory systems, development and conditioning. Behav Brain Res. 2000;110: 25–37. 10802301 10.1016/s0166-4328(99)00182-5

[4] Freeman JH, Stanton ME. Fimbria-fornix transections disrupt the ontogeny of delayed alternation but not position discrimination in the rat. Behav Neurosci. 1991;105: 386–395. 1863360 10.1037//0735-7044.105.3.386

[5] Green RJ, Stanton ME. Differential ontogeny of working memory and reference memory in the rat. Behav Neurosci. 1989;103: 98–105. 2923681 10.1037//0735-7044.103.1.98

[6] Rudy JW, Stadler-Morris S, Albert P. Ontogeny of spatial navigation behaviors in the rat: dissociation of “proximal”- and “distal”-cue-based behaviors. Behav Neurosci. 1987;101: 62–73. 3828056 10.1037//0735-7044.101.1.62

[7] Jablonski SA, Schiffino FL, Stanton ME. Role of age, post-training consolidation, and conjunctive associations in the ontogeny of the context preexposure facilitation effect. Dev Psychobiol. 2012;54: 714–722. 10.1002/dev.20621 22127879 PMC3447086

[8] Pugh CR, Rudy JW. A developmental analysis of contextual fear conditioning. Dev Psychobiol. 1996;29: 87–100. 10.1002/(SICI)1098-2302(199603)29:2<87::AID-DEV1>3.0.CO;2-H 8919089

[9] Rudy JW. Contextual conditioning and auditory cue conditioning dissociate during development. Behavioral neuroscience. 1993;107: 887–91. 10.1037/0735-7044.107.5.887 8280399

[10] Schiffino FL, Murawski NJ, Rosen JB, Stanton ME. Ontogeny and neural substrates of the context preexposure facilitation effect. Neurobiol Learn Mem. 2011;95: 190–198. 10.1016/j.nlm.2010.11.011 21129493 PMC3918451

[11] Langston RF, Ainge JA, Couey JJ, Canto CB, Bjerknes TL, Witter MP, et al Development of the spatial representation system in the rat. Science. 2010;328: 1576–1580. 10.1126/science.1188210 20558721

[12] Wills TJ, Cacucci F, Burgess N, O’Keefe J. Development of the hippocampal cognitive map in preweanling rats. Science. 2010;328: 1573–1576. 10.1126/science.1188224 20558720 PMC3543985

[13] Freeman JH, Steinmetz AB. Neural circuitry and plasticity mechanisms underlying delay eyeblink conditioning. Learn Mem. 2011;18: 666–677. 10.1101/lm.2023011 21969489 PMC3861981

[14] McCormick DA, Thompson RF. Cerebellum: essential involvement in the classically conditioned eyelid response. Science. 1984;223: 296–299. 6701513 10.1126/science.6701513

[15] Berry SD, Thompson RF. Prediction of learning rate from the hippocampal electroencephalogram. Science. 1978;200: 1298–1300. 663612 10.1126/science.663612

[16] Berry SD, Thompson RF. Medial septal lesions retard classical conditioning of the nicitating membrane response in rabbits. Science. 1979;205: 209–211. 451592 10.1126/science.451592

[17] Seager MA, Johnson LD, Chabot ES, Asaka Y, Berry SD. Oscillatory brain states and learning: Impact of hippocampal theta-contingent training. Proc Natl Acad Sci USA. 2002;99: 1616–1620. 10.1073/pnas.032662099 11818559 PMC122239

[18] Solomon PR, Solomon SD, Schaaf EV, Perry HE. Altered activity in the hippocampus is more detrimental to classical conditioning than removing the structure. Science. 1983;220: 329–331. 6836277 10.1126/science.6836277

[19] Nokia MS, Penttonen M, Korhonen T, Wikgren J. Hippocampal theta (3-8Hz) activity during classical eyeblink conditioning in rabbits. Neurobiol Learn Mem. 2008;90: 62–70. 10.1016/j.nlm.2008.01.005 18294872

[20] Hoffmann LC, Berry SD. Cerebellar theta oscillations are synchronized during hippocampal theta-contingent trace conditioning. Proc Natl Acad Sci USA. 2009;106: 21371–21376. 10.1073/pnas.0908403106 19940240 PMC2795537

[21] Wikgren J, Nokia MS, Penttonen M. Hippocampo-cerebellar theta band phase synchrony in rabbits. Neuroscience. 2010;165: 1538–1545. 10.1016/j.neuroscience.2009.11.044 19945512

[22] Hangya B, Borhegyi Z, Szilágyi N, Freund TF, Varga V. GABAergic neurons of the medial septum lead the hippocampal network during theta activity. J Neurosci. 2009;29: 8094–8102. 10.1523/JNEUROSCI.5665-08.2009 19553449 PMC6666051

[23] Berger TW, Alger B, Thompson RF. Neuronal substrate of classical conditioning in the hippocampus. Science. 1976;192: 483–485. 1257783 10.1126/science.1257783

[24] Berger TW, Laham RI, Thompson RF. Hippocampal unit-behavior correlations during classical conditioning. Brain Res. 1980;193: 229–248. 7378816 10.1016/0006-8993(80)90960-9

[25] Berger TW, Rinaldi PC, Weisz DJ, Thompson RF. Single-unit analysis of different hippocampal cell types during classical conditioning of rabbit nictitating membrane response. J Neurophysiol. 1983;50: 1197–1219. 6644367 10.1152/jn.1983.50.5.1197

[26] Berger TW, Thompson RF. Neuronal plasticity in the limbic system during classical conditioning of the rabbit nictitating membrane response. II: Septum and mammillary bodies. Brain Res. 1978;156: 293–314. 101283 10.1016/0006-8993(78)90510-3

[27] Green JT, Arenos JD. Hippocampal and cerebellar single-unit activity during delay and trace eyeblink conditioning in the rat. Neurobiol Learn Mem. 2007;87: 269–284. 10.1016/j.nlm.2006.08.014 17046292 PMC1907365

[28] Hasselmo ME. What is the function of hippocampal theta rhythm?—Linking behavioral data to phasic properties of field potential and unit recording data. Hippocampus. 2005;15: 936–949. 10.1002/hipo.20116 16158423

[29] Hasselmo ME, Schnell E. Laminar selectivity of the cholinergic suppression of synaptic transmission in rat hippocampal region CA1: computational modeling and brain slice physiology. J Neurosci. 1994;14: 3898–3914. 8207494 10.1523/JNEUROSCI.14-06-03898.1994 PMC6576918

[30] Rokers B, Mercado E, Allen MT, Myers CE, Gluck MA. A connectionist model of septohippocampal dynamics during conditioning: closing the loop. Behav Neurosci. 2002;116: 48–62. 11895183

[31] Tóth K, Borhegyi Z, Freund TF. Postsynaptic targets of GABAergic hippocampal neurons in the medial septum-diagonal band of broca complex. J Neurosci. 1993;13: 3712–3724. 7690065 10.1523/JNEUROSCI.13-09-03712.1993 PMC6576440

[32] Goldsberry ME, Elkin ME, Freeman JH. Sensory system development influences the ontogeny of eyeblink conditioning. Dev Psychobiol. 2014;56: 1244–1251. 10.1002/dev.21204 24519393 PMC4119521

[33] Stanton ME, Freeman JH Jr, Skelton RW. Eyeblink conditioning in the developing rat. Behav Neurosci. 1992;106: 657–665. 1503658 10.1037//0735-7044.106.4.657

[34] Harmon TC, Freeman JH. Ontogeny of septohippocampal modulation of delay eyeblink conditioning. Dev Psychobiol. 2015;57: 168–176. 10.1002/dev.21272 25604349 PMC4336210

[35] Goldsberry ME, Kim J, Freeman JH. Developmental Changes in Hippocampal Associative Coding. J Neurosci. 2015;35: 4238–4247. 10.1523/JNEUROSCI.3145-14.2015 25762670 PMC4355197

[36] Ng KH, Freeman JH. Developmental Changes in Medial Auditory Thalamic Contributions to Associative Motor Learning. J Neurosci. 2012;32: 6841–6850. 10.1523/JNEUROSCI.0284-12.2012 22593053 PMC3362655

[37] Berens P. CircStat: A MATLAB Toolbox for Circular Statistics. Journal of Statistical Software. 2009;31.

[38] Kim J, Delcasso S, Lee I. Neural correlates of object-in-place learning in hippocampus and prefrontal cortex. J Neurosci. 2011;31: 16991–17006. 10.1523/JNEUROSCI.2859-11.2011 22114269 PMC3241739

[39] Bragin A, Jandó G, Nádasdy Z, van Landeghem M, Buzsáki G. Dentate EEG spikes and associated interneuronal population bursts in the hippocampal hilar region of the rat. J Neurophysiol. 1995;73: 1691–1705. 7643175 10.1152/jn.1995.73.4.1691

[40] Burke SN, Maurer AP, Nematollahi S, Uprety AR, Wallace JL, Barnes CA. The influence of objects on place field expression and size in distal hippocampal CA1. Hippocampus. 2011;21: 783–801. 10.1002/hipo.20929 21365714 PMC3314262

[41] Le Van Quyen M, Foucher J, Lachaux J-P, Rodriguez E, Lutz A, Martinerie J, et al Comparison of Hilbert transform and wavelet methods for the analysis of neuronal synchrony. Journal of Neuroscience Methods. 2001;111: 83–98. 10.1016/S0165-0270(01)00372-7 11595276

[42] Benjamini Y, Hochberg Y. Controlling the False Discovery Rate: A Practical and Powerful Approach to Multiple Testing. Journal of the Royal Statistical Society Series B (Methodological). 1995;57: 289–300.

[43] Storey JD. A direct approach to false discovery rates. Journal of the Royal Statistical Society: Series B (Statistical Methodology). 2002;64: 479–498. 10.1111/1467-9868.00346

[44] Nokia MS, Sisti HM, Choksi MR, Shors TJ. Learning to learn: theta oscillations predict new learning, which enhances related learning and neurogenesis. PLoS ONE. 2012;7: e31375 10.1371/journal.pone.0031375 22348078 PMC3277498

[45] Inui K, Motomura E, Kaige H, Nomura S. Temporal slow waves and cerebrovascular diseases. Psychiatry Clin Neurosci. 2001;55: 525–531. 10.1046/j.1440-1819.2001.00900.x 11555350

[46] Sato H, Yamamoto K, Kamei C, Shimizu M. Developmental change of EEG in rat from 4th to 16th week. Experientia. 1979;35: 879–880. 477837 10.1007/BF01955125

[47] Brown KL, Freeman JH. Extinction, reacquisition, and rapid forgetting of eyeblink conditioning in developing rats. Learn Mem. 2014;21: 696–708. 10.1101/lm.036103.114 25403458 PMC4236410

